# Certificated Dispensers and the Insurance Bill

**Published:** 1911-11-25

**Authors:** 


### The Editor's Letter Box.

### CERTIFICATED DISPENSERS AND THE INSURANCE BILL.

*To the Editor of* The Hospital.

Sir,—I am glad to find from the letter of "Collegia" in your last issue that the Association of Certificated Dispensers is taking action to protect the interests of those dispensers who will be affected by the National Insurance Bill. At the same time, it is to be hoped that dispensers will write to their respective Members of Parliament and ask them to propose or support the amendment suggested on page 137 of The Hospital of November 4, which will at least protect our existing interests. The Report stage of the Bill will be reached in a few days, and unless the clause is amended then, it will be too late. Members should also be reminded of the provisions of the Pharmacy and Poison Act, 1908, for the registration of apothecaries' assistants as pharmacists without examination, and it should be pointed out to them that the Pharmaceutical Society are at the present time formulating by-laws for the registration of Colonial pharmacists, but are taking no action with regard to apothecaries' assistants, which means that unless we obtain recognition under the Insurance Bill, as a class we are doomed to extinction. Under these circumstances it behoves each one to do all he can for the common good, and it seems to me that this can but be accomplished by the appeal of each individual dispenser to their local Member of Parliament. Of course, if the Association of Certificated Dispensers has an official amendment, that should be supported. Failing that, I ask for the support of dispensers generally to the amendment on page 137 of your issue of November 4.

With regard to the letter signed "Patiens" in your issue of the 18th inst., I can only say that the statement imputed to me "that the Association of Certificated Dispensers and its Secretary have been asleep" is not strictly accurate. It is true I contrasted the action of pharmacists with that of the certificated dispensers of the Apothecaries' Hall, and said "the Association of the members and the Secretary of the Society have been apparently asleep." I think this is justifiable. There has been no action by the Association of Certificated Dispensers, and the Secretary of the Society (meaning thereby the Society of Apothecaries) has, as far as I know, taken no action. Anyhow, neither the Association of Certificated Dispensers nor the Secretary of the Society of Apothecaries has taken the action that has been taken by the Secretary of the Pharmaceutical Society and pharmacists generally. With regard to the Secretary of the Association of Certificated Dispensers, I did not mention him, and am sorry that my remarks should be construed as reflecting on him, for I know how energetically he has worked and how much the thanks of all of us are due to him. My object has been to induce others to come forward and do their share at this critical juncture. Next week the Report stage of the Bill will be reached, and unless an amendment in our favour is then made it will be too late. I therefore think that every Apothecaries' assistant throughout the United Kingdom will write to their local member of Parliament and ask him to support the amendment of Clause 14 (5) (iii.) on the lines indicated on p. 137 of your issue of November 4. Pharmacists are making strenuous efforts to prevent any alteration of the clause, so it behoves each one of us to do what we can for our common good.—Yours faithfully,

An Apothecaries' Assistant.

### MALVERN HOSPITAL.

*To the Editor of* The Hospital.

